# Assist me or replace me? Uncovering the influence of AI awareness on employees’ counterproductive work behaviors

**DOI:** 10.3389/fpubh.2024.1449561

**Published:** 2024-10-03

**Authors:** Shizhen Bai, Xiaoxue Zhang, Dingyao Yu, Junru Yao

**Affiliations:** ^1^School of Management, Harbin University of Commerce, Harbin, China; ^2^China Academy of Civil Aviation Science and Technology, Beijing, China

**Keywords:** artificial intelligence awareness, conservation of resource theory, counterproductive work behaviors, emotional exhaustion, psychological contract, psychological contract breach

## Abstract

**Objective:**

Drawing on the conservation of resources theory (COR), the research aims to reveal the influence of artificial intelligence (AI) awareness on employees’ mental health and behaviors, particularly examining whether and how employees’ AI awareness impacts their counterproductive work behaviors (CWB) in human-intelligence collaborations.

**Methods:**

Data was collected from 327 Chinese employees who collaborated with AI in sales, manufacturing, logistics, and other industries. The measurement instruments included scales for AI awareness, psychological contract (PC), emotional exhaustion (EE), and counterproductive work behavior (CWB). We used Hayes’s PROCESS macro to analyze the data.

**Findings:**

AI awareness had a significant positive impact on CWB (*β* = 0.448, *p* < 0.01). PC and EE play a role as partial mediators in the relationship between AI awareness and CWB. The mediating pathways consist of three sequences: “AI awareness → PC → CWB,” “AI awareness → EE → CWB” and “AI awareness → PC → EE → CWB,” with the respective contributions to the overall effect amounting to 8.04, 18.53, and 4.91%.

**Discussion:**

Our research contributes to the study of AI in the management field by elucidating the relationship between AI awareness and CWB, as well as the mediating mechanisms of this relationship, which enriches the literature on CWB and expands the understanding of the associations between AI and CWB.

## 1 Introduction

With the development of the Fourth Industrial Revolution, AI is increasingly used in various industries due to its outstanding advantages in reducing costs, increasing efficiency, fostering innovation, and spurring growth (1–3). For example, the education sector is using AI technology for intelligent assignment correction (4, 5). In the manufacturing field, AI technology automates work processes. Within the service industry, AI is increasingly being integrated into hospitality, catering, and tourism enterprises as robots (6). Additionally, the medical industry employs AI to assist with patient treatment (7–10).

The rapid advancement of AI applications is revolutionizing the boundaries of organizations and the careers of their employees. As a result, employees are exposed to an uncertain work environment (11), affecting their personal feelings and career development (12). SDG 8, one of the 17 Sustainable Development Goals proposed by the United Nations in 2015, mentions the need to “promote inclusive and sustainable economic growth, employment and decent work for all.” Several previous studies have found that AI adoption may potentially threaten employees, resulting in adverse psychological effects, such as a sense of insecurity about jobs that may be replaced (13). Moreover, High level of AI awareness could lead to negative behaviors, including work withdrawal and staff turnover (2, 14). The effects may counter the goal of “safe and secure working environments” advocated by SDG8 and undermine employee well-being. However, research into employees’ attitudes and behavioral responses to AI is still in its initial stages (15).

In management research, employees’ concerns about the introduction of AI-related technologies are referred to as AI awareness (16). AI awareness is the degree to which employee perceives that AI-related technologies will harm the future of their career (16). Researchers have already found that AI awareness can significantly impact employee behavior in the hospitality sector. For example, Li et al. (2) empirically demonstrated that high level of AI awareness can significantly increase employees’ turnover intention. Zhao et al. (17) proposed a positive correlation between AI awareness and organizational deviance among service staff. Teng et al. (14) found that AI awareness can lead hotel staff to withdraw from their work. Nevertheless, in a more general context, whether this similar negative behavior will also exist in other industries and the mechanism behind this relationship have piqued our interest.

Counterproductive work behavior (CWB) is another kind of negative behavior, referring to unethical actions by employees that damage or are intended to damage the organization’s legitimate interests (e.g., production deviations, interpersonal avoidance). CWB is perceived as highly unfavorable to the organization and can cause damage to its reputation, property, and other resources (18, 19). Despite the initial findings of current scholars, there remain research gaps on whether employee AI awareness will lead to CWB, such as employee resistance and retaliation against the organization. The underlying influence mechanism has yet to be disclosed. Therefore, this study focuses on two issues:

**RQ1:** *Does employee AI awareness influence CWB, and if so, how?*

**RQ2:** *What are the mediating mechanisms between the two?*

We introduce the COR theory to construct our research framework to fill the aforementioned research gaps. The COR theory posits that individuals prioritize the acquisition and retention of resources, and strive to avoid resource loss. The consequences of resource loss are considered more significant than those of resource gain (20). When companies introduce technologies such as AI, employees fear being replaced (13). This fear prompts them to acknowledge the possibility of losing their jobs or facing significant challenges in maintaining their positions. Such scenarios represent atypical and significant resource loss. In accordance with COR theory, individuals tend to exhibit aggressive behavior when their perceived resources are exhausted. Aggressive behavior is a common manifestation of CWB within organizations. CWB encompasses actions by employees that either harm or are intended to harm the legitimate interests of the organization, potentially leading to a range of negative impacts. Examples of CWB include deviating from production standards, engaging in conflicts with coworkers, and acts of sabotage (18, 21). Therefore, this study suggests that AI awareness will likely trigger employees’ CWB.

Individual’s AI awareness can lead to the depletion of his resources, triggering negative emotions like emotional exhaustion (EE). EE intensifies the depletion of resources and raises the probability of engaging in subsequent negative behaviors, including CWB. In other words, EE can positively affect CWB (22). Therefore, based on COR, we suggest the possibility that heightened AI awareness may cause employees to experience emotional exhaustion, which, in turn, may lead them to retaliate against their organization through CWB (23).

The psychological contract (PC) is an exchange relationship based on reciprocity (24, 25), a tacit agreement between the employee and the employer. It is critical for employee engagement and performance (26). Employees with stronger PC are more engaged in their work and less inclined to get involved in the CWB. However, AI awareness makes employees feel threatened by the potential replacement, leading them to perceive the organization as betraying their contracts. Consequently, employees experience a significant cognitive dissonance. According to the COR theory, the loss of personal resources is more pronounced, which may exacerbate CWB. Therefore, we propose that heightened AI awareness might lead to a decrease in employees’ perceived PC, which could potentially elicit CWB. The perceived loss of resources, stemming from this decrease, triggers negative emotions and depletes employees’ psychological resources. According to the COR theory, this depletion of psychological resources may result in EE, which could, in turn, prompt severe behavioral responses, such as CWB, as a means of protecting existing resources. Therefore, we predict that PC and EE may serve as sequential mediators in the relationship between AI awareness and CWB.

## 2 Theoretical background and hypotheses development

### 2.1 Conservation of resource theory

Conservation of Resource (COR) Theory explains the causes of stress and individual behavior by focusing on resource acquisition or consumption. It is widely used in research related to human resource management and can provide a research framework for understanding how resource changes and stress impact work outcomes. This theory suggests that individuals strive to acquire, retain, and build what is valuable and important to them, referred to as resources (27). Stress occurs when an individual perceives that their resources are (1) in danger of being lost, (2) lost, or (3) unrewarded for their efforts. The individual will then take action to alleviate pressure (20, 27). The COR theory encompasses a series of principles and inferences, providing a more comprehensive explanation of the causes and impacts of individual behavior. For example, resource losses are more easily perceived and noticed than resource gains, thus having a greater impact. People can preserve resources by investing in them; those with fewer resources are more susceptible to the effects of resource depletion compared to those who are resource-rich. The depletion of resources can put individuals into a defensive mode, increasing the likelihood of aggressive behavior (20, 28, 29).

Our study aimed to clarify the relationship between AI awareness and CWB. Past research has identified AI awareness as a source of stress (13). The COR theory can be employed to explain the causes and effects of stress formation in different contexts (20, 28). COR theory suggests that stress generation leads to negative employee emotions and EE (30), prompting individuals to seek supplemental resources or perhaps even engage in drastic negative behaviors (20, 31). CWB represents a typical negative employee outcome (32). Therefore, COR theory can assist us in understanding the relationship between a stressor such as AI awareness and a negative employee outcome such as CWB.

Additionally, the COR theory posits that the occurrence of a resource loss can lead to subsequent losses, creating a chain reaction that intensifies the negative impact of the initial loss (20). Therefore, as a source of strain, AI awareness could trigger a cascade of events that exacerbate resource depletion, culminating in detrimental employee outcomes (CWB). Consequently, we propose the existence of mediating variables in the form of a chain reaction between AI awareness and CWB. In conclusion, the COR theory is particularly well-suited to explaining our research model due to its emphasis on the dynamics of resource loss and its aftermath.

### 2.2 AI awareness and CWB

Counterproductive work behavior (CWB) refers to actions performed by employees that damage or intend to damage their organization’s legitimate interests. These behaviors are spontaneously generated by employees (32). This immoral behavior, which is spontaneous, violates organizational or social norms and can lead to various adverse effects on the organization, such as production deviation and destruction (18, 21). CWB is divided into two categories: CWB-organizational (CWB-O), such as production deviations and sabotage, and CWB-interpersonal (CWB-I), including abuse and other such acts (32, 33). This study focuses on CWB-O. Given the severe implications of CWB on organizational interests, researchers have investigated multiple facets of its underlying causes to mitigate its occurrence. Internal personal factors, including feelings of insecurity (34), specific personality traits (35, 36), and varying levels of intrinsic motivation (37, 38) can serve as triggers for CWB. Moreover, external factors such as leadership styles (39, 40), organizational constraints (41), and the quality of interpersonal relationships both within the organization and with customers (42, 43), can also impact CWB. There has been very little research into AI awareness as a causal factor in CWB, and the existing studies have primarily been conducted within the hospitality service industry (44). There is a notable lack of research on AI awareness as a trigger for CWB in a broader industry context. Our study aims to fill this research gap.

AI awareness poses a risk of replacement for employees. This feeling threatens their resources, such as employment opportunities, working conditions, and job content (20, 27). Consequently, AI awareness represents employees’ concerns about their future job prospects due to the use of AI, which is a negative psychological state. The higher the level of AI awareness, the stronger the perception of harm brought by AI. A functional identification framework for AI was developed by Selenko et al. (45), which categorizes the impact of AI use on employees into three types (12, 46). Following this classification, we also divide AI awareness into three categories. The first type pertains to the threat posed by AI providing technical support or supplementing existing human work. The adoption of AI leads to changes in how employees perform their tasks, potentially requiring them to abandon or adapt their old skills and learn new ones (47). These changes can make employees feel that they have lost their resources, such as their original work abilities and the stability of their job content. Consequently, employees may feel threatened. The second type involves the sense of threat created by AI replacing human tasks. As AI outperforms humans in many aspects (48), employees face the risk of job displacement or unemployment. The loss of this resource is more threatening than the first type. The third type pertains to the new human work tasks generated by AI. In this case, employees do not lose their jobs but must acquire new skills. Like the first type, people may lament their original work and current status (49). Current research on AI awareness primarily focuses on service industries such as hospitality, catering and nursing. Tan et al. (50) and Teng et al. (14) have investigated the impact of hotel employees’ AI awareness on employee behavior from the perspectives of individual competitiveness and work withdrawal, respectively; Liang et al. (51) examined the influence of AI awareness on innovative behavior among service industry employees; Kwak et al. (52) researched the effect of AI awareness on the behavior of employees in the nursing industry; and Flavián et al. (53) conducted a correlation study on the technological readiness of employees in the banking and financial industry in relation to AI awareness.

According to COR theory, AI awareness is considered one of the sources of work-related stress (13, 14). AI awareness makes employees feel that their resources may be robbed by AI, engendering feelings of loss and stress. Such feelings can, in turn, result in negative attitudes or behaviors, including CWB. Consequently, there is a positive correlation between AI awareness and CWB. We assume employees’ AI awareness can spark their CWB from the perspective of COR theory. The reasons are as follows.

First, AI awareness creates a perception of uncertainty among employees that AI may eventually replace their work (16). This substitution could lead to unemployment, possibly causing consequences that will disrupt their lives and result in resource loss. This risk is beyond the control of employees. Therefore, they tend to attribute it to the organization as a result of its AI adoption, making it impossible to provide them with fair and satisfying work. The COR theory suggests that people must invest resources to prevent resource loss. Consequently, employees may engage in CWB, such as breaking organizational rules, to retaliate against the organization and protect their psychological resources (54). Therefore, the stronger the AI awareness, the greater the risk of depletion of an employee’s psychological resources. According to COR theory, this heightened risk may lead the employee to engage in coping strategies in order to maintain psychological balance, thus preventing the loss of psychological resources. Consequently, there is a greater likelihood that the employee will resort to CWB due to these coping mechanisms (55). It is considered a method of acquiring and replenishing resources. In other words, when perceiving resource loss threats from AI, employees offset the loss of resources by increasing their psychological resources through CWB.

Second, according to the COR theory, individuals enter an aggressive defense mode when they perceive their resources being depleted or exhausted (20). With the increasing AI awareness, employees perceive a competitive or even hostile relationship with AI. They may face replacement (56) or need to abandon their old skills and learn new ones to adapt to the change (47). This kind of stress drains the employee’s psychological resources. As stress increases, AI awareness also grows, leading to the perception of psychological resource depletion. CWB can be viewed as a form of protest behavior, thereby defending against perceived unfairness or other influences (57). In response, employees tend to engage in CWB as an aggressive defense mode to mitigate the expanding loss of resources.

Third, the uncertainty of future careers due to AI awareness can have adverse psychological effects on employees, including pessimism, cynicism, burnout, emotional exhaustion, and insecurity (2, 51, 58). Previous scholars have demonstrated the positive impacts of negative emotions on CWB (59, 60). Employees’ AI awareness leads them to experience multiple negative emotions, thus making them more inclined to be involved in CWB. Thus, we propose that:

**H1:** *AI awareness can significantly intensify CWB.*

### 2.3 Mediating role of EE

EE refers to being emotionally overwhelmed, which depletes one’s emotional resources (61). According to COR, EE is a representative symptom of resource loss. Some scholars have empirically validated that AI awareness can trigger EE (14, 51). Employees’ AI awareness is a source of stress and can be considered a job-related demand (62). Addressing this stress and demand may entail psychological costs and result in the depletion of resources (63, 64). Chronic stress can exhaust employees’ psychological resources over time (65). Consequently, if no interventions are implemented to reduce stress, and employees lack adequate resources for coping, AI awareness may persistently deplete these resources, potentially culminating in employees experiencing EE (66, 67).

EE has been shown to negatively affect employees’ psychology and behavior (68, 69). Therefore, we believe there is a link between EE and employees’ CWB. First, EE depletes employees’ mental and emotional resources, leaving them with insufficient reserves to cope with stress (70). This, in turn, diminishes their ability to regulate their behavior on the job, making it more difficult for them to stay on top of tasks, and increases the likelihood of CWB. Golparvar (71) posited that CWB may be a maladaptive coping strategy. For instance, in the hotel industry, employees may be unable to focus on their work due to EE, which increases the likelihood of work withdrawal and CWB (14, 44). Second, similar to the Matthew effect, the COR theory suggests that when individuals face a loss of resources, they become more susceptible to future losses (20). Since AI awareness can reduce employees’ psychological resources, emotionally exhausted employees are more likely to develop CWB due to insufficient resources to regulate and normatively constrain their behavior. Jia et al. (72) indicates that in the computer industry, employees’ increased sense of job insecurity and EE has led to more instances of workplace deviance behavior. Third, in response to EE caused by stress, employees may express their negative emotions through various means. Individuals are likely to protect their limited resources through CWB (22). If employees lack the resources to cope with EE, they may exhibit CWB. Chen et al. (73) used data from both China and the United States to demonstrate that employees in manufacturing firms, due to EE, exhibited more aggressive forms of CWB. This aligns with the COR theory, which posits that as an individual’s resources become depleted, their behavior may become more aggressive. In summary, employee EE is a mechanism linking AI awareness to CWB. So, we hypothesize:

**H2:** *EE mediates the relationship between AI awareness and CWB.*

### 2.4 Mediating role of PC

The PC refers to an employee’s perception of their reciprocal exchange relationship with their employer (24, 25). There are two categories of PC: transactional and relational contracts (74). The transactional contract is materialistic and focuses on short-term benefits. In contrast, the relational contract denotes a mutually beneficial relationship between the company and its staff for mutual development (75). Consequently, the relational contract tends to favor both the organization and the employee. Millward and Hopkins (76) also suggested that organizations can achieve greater profitability by focusing on relational aspects. Therefore, the PC mentioned in this paper refers to the relational contract.

According to COR theory, AI awareness among employees may lead to a perceived loss of resources. AI awareness often originates from organizational initiatives to implement AI. Consequently, it can diminish the perceived PC between employees and the organization, which, in turn, may trigger a spectrum of negative emotions and behaviors, including CWB. The reasons are as follows.

The creation and exacerbation of AI awareness can reduce the perceived PC between employees and their organizations. On the one hand, when AI is introduced into organizations, employees’ AI awareness may lead them to fear that their organization might replace them with AI. This fear could result in the jobs promised by the organization and their career plans within the business disappearing (77). Such concerns can significantly deplete the psychological resources of the employees, which, in turn, may lead to a decrease in their perception of the PC between them and the organization (78). Second, based on the COR theory, employees’ AI awareness is recognized as a work-related stressor. The creation and exacerbation of this awareness can lead employees to experience more pronounced psychological impacts and a noticeable sense of stress. The PC between employees and organizations suggests that employees believe their employer should care about their development and well-being. This belief fosters a closer, longer-lasting, and more profound connection with the organization (79). However, AI awareness diminishes the above-mentioned psychological expectations of the employee, creating a sense of PC violation.

COR theory posits that individuals are motivated to safeguard their existing resources and to avert potential resource loss. When employees experience a perceived breach of the PC by the organization, the intensity of their sense of betrayal and the perceived loss of resources is often proportional to the strength of the PC. In response, a defensive mechanism, exemplified by CWB, is more likely to manifest. On the one hand, a weakened perception of the PC diminishes employees’ sense of belonging and commitment to the organization (80, 81), leading them to become indifferent toward the organization’s interests and resulting in CWB. On the other hand, the depletion of resources due to the weakened PC increases the probability of employees facing resource scarcity. Consistent with COR theory, this predicament will likely provoke more severe retaliatory actions, including CWB, as employees strive to protect their dwindling resources. Therefore, we hypothesize:

**H3:** *The PC mediates the relationship between AI awareness and CWB.*

### 2.5 Chain mediating roles of PC and EE

**Figure 1:**
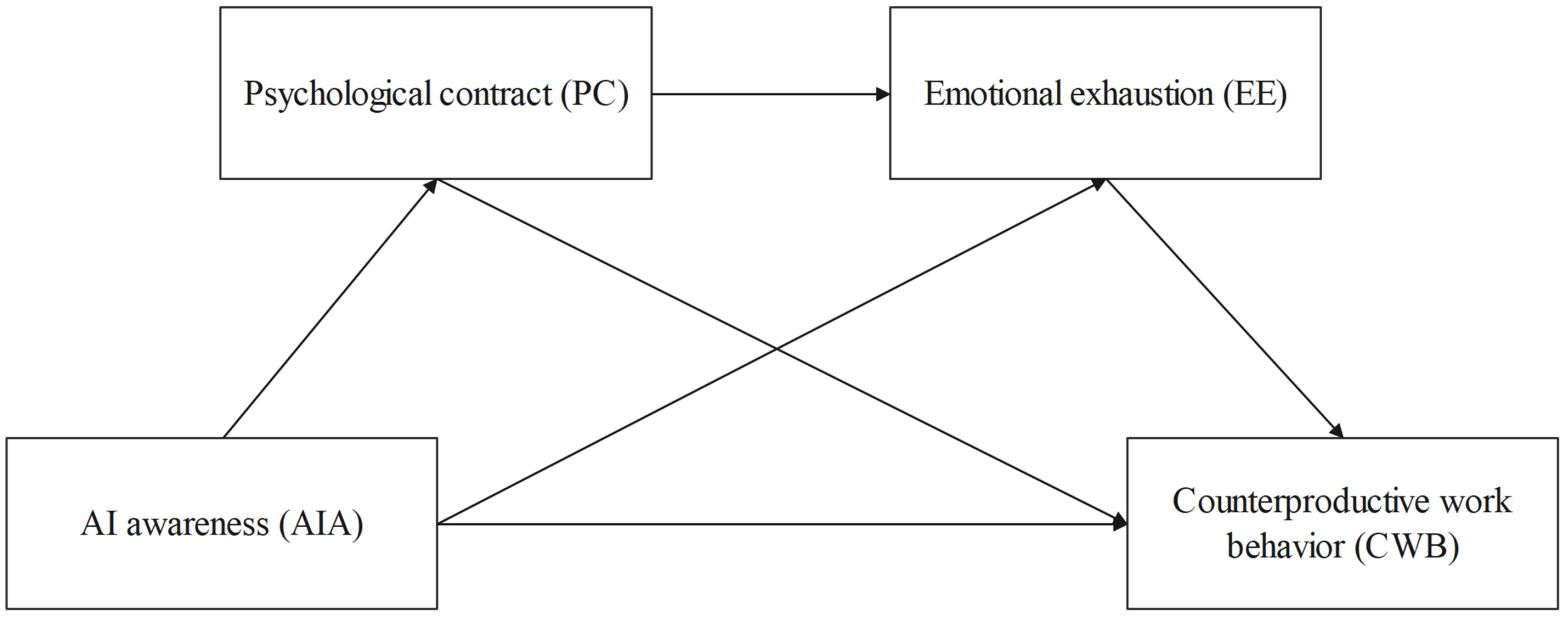
Theoretical model.

Based on the analysis, PC and EE may act as sequential mediators in the relationship between AI awareness and CWB. The prior analysis suggests that the creation and exacerbation of AI awareness led to a decrease in employees’ perceptions of PC, which may make employees feel that the firm has violated the PC between them (58, 82). Therefore, AI awareness diminishes employees’ PC perceptions. As employees’ perceptions of betrayal due to contract breaches increase, their sense of organizational belongingness diminishes (80). This results in more negative emotions and stress (83), further depleting employees’ resources. In the absence of resources to compensate, the persistence of this state has the potential to deplete employees’ psychological and emotional resources, leading to EE (84, 85). The presence of EE leaves employees with no spare resources to regulate their behavior at work or even to express their dissatisfaction more intensely, leading to CWB. In summary, we argue that AI awareness decreases employees’ PC, and the decrease in PC further depletes psychological resources, increases EE, and ultimately triggers employees’ CWB. Therefore, we hypothesize that:

**H4:** *PC and EE assume the role of chain mediators between AI awareness and CWB.* The figure illustrates the theoretical model (see Figure 1).

## 3 Methodology

### 3.1 Samples

Our study examines the influence of AI awareness on employee emotions and behaviors during human-intelligence collaboration. Thus, the target participants should be those with more contact with AI at work. Drawing on previous research (86–88), we selected eight industries highly affected by AI for our study, such as logistics, sales and manufacturing. We limited the sample to eight industries that have more contact with AI (86). This approach enables the sample to be more representative.

We collected data through questionnaires. We recruited participants and collected data on, which scholars widely use (51). The website collected basic personal information from participants, including the industries in which they worked. We implemented restrictions to ensure that only those within specific industry ranges could access the questionnaire. Additionally, we recorded the participants’ industry information within the questionnaire to further validate and confirm the relevance of the sample. The respondents are employees who need to collaborate with AI at work. In addition to limiting the industries, we also set up a screening item (i.e., “Has your company introduced AI technology in your work? Do you need to use AI in your current job?”) to further identify whether participants need to collaborate with AI technologies in their work. Only those who chose “YES” were allowed to continue; if the answer was No., the questionnaire would be closed. We provided material incentives to participants through Credamo, clarifying before the questionnaire began that the data collected was for the exclusive purpose of the study and that their confidentiality would be ensured. A total of 450 questionnaires were distributed, with 89 being excluded at the filtering question stage, leaving 361 questionnaires for analysis. Among the submitted questionnaires, 31 were completed in less than 1 min, and 3 failed to pass the attention check, resulting in a final sample of 327 valid questionnaires. The validity rate was 72.7%. The gender distribution among the participants is relatively balanced, with a higher proportion from the eastern region, accounting for 51.4%, followed by the central region at 28.7%, and the western region having the lowest proportion at 19.9%. This distribution aligns with the economic development levels of various regions in China. The specific demographic information of the subjects is in Table 1.

**Table 1:** Demographic information.

Feature	Category	Frequency	Percentage
Gender	Male	166	50.8
	Female	161	49.2
Age	Less than 20 years old	16	4.9
	21–30 years old	142	43.4
	31–40 years old	101	30.9
	41–50 years old	30	9.2
	51 years old and above	38	11.6
Education	Junior high school and blow	2	0.6
	High school	26	8.0
	College	228	69.7
	Master’s degree and above	71	21.7
Tenure	Less than 1 year	37	11.3
	1–3 years	77	23.5
	4–6 years	70	21.4
	7–9 years	63	19.3
	More than 10 years	80	24.5
Monthly income	Less than 3,000	29	8.9
	3,000–5,000	38	11.6
	5,000–8,000	85	26.0
	8,000–10,000	56	17.1
	More than 10,000	119	36.4
Area	Eastern Province	168	51.4
	Central Province	94	28.7
	Western Province	65	19.9
Industry	Logistics	16	4.9
	Sales	30	9.2
	Education	52	15.9
	Administration & office support	36	11.0
	Manufacturing	80	24.5
	Agriculture	13	4.0
	Construction	21	6.4
	Service	79	24.2

### 3.2 Measurement

Well-established scales widely accepted by academics were used in our study. A 5-point Likert scale was adopted for all questions except the control variables. The mean score of the items was employed to ascertain the variable’s value. All the scores range from 1 (strongly disagree) to 5 (strongly agree). We translated the scale from English to Mandarin following Brislin’s suggestion (89). The accuracy of the translation was also confirmed by consulting three language scholars and an industry expert. Additionally, a pre-test involving 50 individuals was conducted to validate the questionnaire’s effectiveness, and modifications were made to some of the questions translated into Chinese to prevent ambiguity.

*AI awareness (AIA).* We used four items Brougham and Haar (16) developed to measure employee AI awareness. The scale consists of four items, including “I am personally worried that what I do now in my job will be able to be replaced by AI.” A higher score indicates a stronger perception of AI awareness, meaning that the employee has a greater concern about being replaced by AI. The Cronbach’s alpha value was 0.889. *Emotional exhaustion (EE)*. Referring to Maslach and Jackson (90), nine items were adopted to measure the employees’ EE. The scale comprises nine items, such as “I feel frustrated by my job.” A higher score indicates a stronger perception of EE. The Cronbach’s alpha was 0.905. *Psychological contract (PC)*. Nine items adapted from Raja et al. (91) were used to measure the PC. The PC scale consists of nine items, including “I have a reasonable chance of promotion if I work hard.” A higher score indicates a stronger perception of PC with the organization among employees. The Cronbach’s alpha was 0.912. *Counterproductive work behavior (CWB)*. CWB was measured using a scale developed by Dalal et al. (92). The scale contained six items, such as “Did not work to the best of my ability. “A higher score indicated a greater degree of CWB among employees. The Cronbach’s alpha coefficient was 0.863.

Among the scales, the Cronbach’s alpha coefficient for PC and EE exceeds 0.9, indicating high internal consistency and suggesting that the measurement results are reliable. To some extent, the high degree of consistency among the questionnaire items aligns with our theoretical framework, which emphasizes the constructs’ intrinsic unity. An alpha value this high may also imply a high degree of interrelatedness among the items, which could lead to multicollinearity issues and affect the accuracy of statistical analyses. To assess the potential for multicollinearity, we conducted a Variance Inflation Factor (VIF) analysis and found that the VIF values for all variables were far below 5, indicating that multicollinearity is within an acceptable range. All the scale items are shown in Table 2. Chin (93) suggests that most of the loadings on the question items should be at least 0.60 and ideally at 0.70 or above. All factor loadings on our scale were above 0.6, which is consistent with the requirements. Table 3 shows the factor loadings, CR, and AVE.

**Table 2:** Scale items used in study.

Scale	Items
AI awareness (AIA)	I think my job could be replaced by AI.
	I am personally worried that what I do now in my job will be able to be replace by AI.
	I am personally worried about my future in my organization due to AI replacing employees.
	I am personally worried about my future in my industry due to AI replacing employees.
Psychological contract (PC)	I expect to grow in this organization.
	I feel part of a team in this organization.
	I have a reasonable chance of promotion if I work hard.
	To me working for this organization is like being a member of a family.
	The organization develops/rewards employees who work hard and exert themselves.
	I expect to gain promotion in this company with length of service and effort to achieve goals.
	I feel this company reciprocates the effort put in by its employees.
	My career path in the organization is clearly mapped out.
	I am motivated to contribute 100% to this company in return for future employment benefits.
Emotional exhaustion (EE)	I feel emotionally drained from my work.
	I feel used up at the end of the workday.
	I feel fatigued when I get up in the morning and have to face another day on the job.
	Working with people all day is really a strain for me.
	I feel burned out from my work.
	I feel frustrated by my job.
	I feel I am working too hard on my job.
	I feel like I am at the end of my rope.
	Working with people directly puts too much stress on me.
Counterproductive work behavior (CWB)	Did not work to the best of my ability.
	Spent time on tasks unrelated to work.
	Criticized organizational policies.
	Took an unnecessary break.
	Worked slower than necessary.
	Spoke poorly about my organization to others.

**Table 3:** Factor loadings, CR, and AVE.

Constructs and items	Factor loadings	CR	AVE
AI awareness (AIA)		0.890	0.671
AIA1	0.75		
AIA2	0.85		
AIA3	0.858		
AIA4	0.813		
Psychological contract (PC)		0.913	0.538
PC1	0.719		
PC2	0.811		
PC3	0.745		
PC4	0.746		
PC5	0.711		
PC6	0.705		
PC7	0.713		
PC8	0.722		
PC9	0.726		
Emotional exhaustion (EE)		0.905	0.516
EE1	0.713		
EE2	0.697		
EE3	0.718		
EE4	0.707		
EE5	0.793		
EE6	0.669		
EE7	0.753		
EE8	0.691		
EE9	0.717		
Counterproductive work behavior (CWB)		0.865	0.516
CWB1	0.692		
CWB2	0.730		
CWB3	0.698		
CWB4	0.732		
CWB5	0.727		
CWB6	0.729		

*N* = 327. CR, construct reliability; AVE, average variance extracted.

## 4 Results

### 4.1 Common method bias

In order to control common method bias, we conducted a pre-test before data collection. Additionally, parts of the questions were adapted to enhance the project’s comprehensibility. Data collection was performed anonymously, and participants were guaranteed confidentiality and legality. Afterward, we conducted Confirmatory factor analyses (CFAs) to ensure the discriminant validity of the 4-factor model was the best compared to other models. The results of the model fit according to AMOS support our idea, which means that the model for this study had good discriminant validity. The confirmatory factor analyses of four types of factor combinations are in Table 4.

**Table 4:** The confirmatory factor analysis.

Model	Combination	*χ*^2^/df	IFI	TLI	CFI	RMSEA	sRMR
One-factor model	AIA + EE + PC + CWB	6.015	0.660	0.630	0.658	0.124	0.1057
Two-factor model	AIA + EE + CWB, PC	3.775	0.812	0.795	0.811	0.092	0.0705
Three-factor model	AIA + CWB, EE, PC	2.669	0.888	0.877	0.887	0.072	0.0556
Four-factor model	AIA, EE, PC, CWB	1.989	0.934	0.927	0.934	0.055	0.0492

### 4.2 Correlation analysis

The correlation between the core variables is in Table 5. We can see from the correlation coefficients that the core variables are significantly correlated. Furthermore, all the correlation directions align with what we assumed, which initially validates our idea.

**Table 5:** Means, standard deviations, and correlations.

	*M*	SD	AIA	PC	EE	CWB
AIA	2.927	1.090	(0.819)			
PC	4.015	0.638	−0.433**	(0.733)		
EE	2.673	0.818	0.608**	−0.529**	(0.718)	
CWB	2.645	0.812	0.626**	−0.444**	0.576**	(0.718)

*N* = 327. The values in parentheses represent the square roots of AVE. AIA, AI Awareness; PC, Psychological Contract; EE, Emotional Exhaustion; CWB, Counterproductive Work Behavior. **p < 0.01 (two-tailed).

### 4.3 Hypothesis testing

We used gender, age, educational background, income, tenure, and geographic region as control variables to test the models by regression. Scholars frequently incorporate control variables into their models when examining human psychology and behavior to ensure the reliability of the findings (94). On one hand, disparities in participants’ gender, age, and educational background may affect their cognition, psychological state, and behavior (95–97). Consequently, we have considered these factors as control variables in our study. On the other hand, the current deployment of AI is not uniform, and regional differences may result in variations in employees’ perceptions of AI. Hence, we have also included regional factors among the control variables to minimize the potential for confounding results attributable to regional discrepancies. In particular, we include industry as a control variable to examine whether the negative effects of AI awareness are widespread across different industries, as previously mentioned.

We used SPSS and macro PROCESS to test the hypotheses. The results are presented in Table 6. Initially, when analyzing the primary associations in Model 4, AI awareness is positively and significantly associated with CWB, with the control variables taken into account, with a coefficient of *β* = 0.448 (*p* < 0.01). Therefore, Hypothesis 1 was supported. Meanwhile, as can be seen from Models 1 and 2, the association between AI awareness and PC (*β* = −0.224, *p* < 0.01) as well as EE (*β* = 0.444, *p* < 0.01) was also significant. Moreover, PC is negatively and significantly associated with both EE and CWB, with impact coefficients of −0.417 and −0.258, respectively, as shown in Models 3 and 5. As can be seen from Model 7, with the control variables unchanged, the addition of the two mediator variables, PC and EE, to the main model diminishes the association between AI awareness and CWB from 0.448 in Model 4 to 0.307 in the combined model. This indicates that PC and EE moderate the relationship between AI awareness and CWB, suggesting that the mediating role is observed.

**Table 6:** Regression analysis results.

	M1	M2	M3	M4	M5	M6	M7
Variables	PC	EE	EE	CWB	CWB	CWB	CWB
AIA	−0.224^***^	0.444^***^	0.350^***^	0.448^***^	0.390^***^	0.320^***^	0.307^***^
	(−7.558)	12.965	(10.089)	(13.598)	(11.192)	(8.234)	(7.875)
PC			−0.417^***^		−0.258^***^		−0.159^***^
			(−6.903)		(−4.255)		(−2.515)
EE						0.287^***^	0.237^***^
						(5.561)	(4.319)
Gender	0.112^*^	−0.123^*^	−0.077	−0.174^**^	−0.145^**^	−0.139^**^	−0.127^*^
	(1.763)	(−1.689)	(−1.120)	(−2.479)	(−2.114)	(−2.058)	(−1.897)
Age	0.081^*^	0.034	0.068	0.077	0.098	0.068	0.082^*^
	(1.711)	(0.626)	(1.327)	(1.479)	(1.917)	(1.350)	(1.643)
Education	0.085	−0.106	−0.071	0.027	0.049	0.057	0.066
	(1.450)	(−1.568)	(−1.115)	(0.414)	(0.769)	(0.919)	(1.058)
Tenure	−0.003	−0.057	−0.058	−0.102^**^	−0.103^**^	−0.086^*^	−0.089^**^
	(−0.066)	(−1.203)	(−1.314)	(−2.258)	(−2.333)	(−1.982)	(−2.073)
Income	0.057^*^	0.010	0.034	−0.001	0.014	−0.004	0.006
	(1.942)	(0.296)	(1.063)	(−0.020)	(0.440)	(−0.013)	(0.194)
Area	−0.036	0.049	0.034	0.015	0.006	0.001	−0.003
	(−0.882)	(1.048)	(0.780)	(0.328)	(0.127)	(0.017)	(−0.059)
Industry	−0.015	0.017	0.011	0.037^**^	0.033^**^	0.032^**^	0.030^**^
	(−1.073)	(1.064)	(0.722)	(2.327)	(2.129)	(2.099)	(2.011)
Constant	3.955^***^	1.771^***^	3.422^***^	1.417^***^	2.437^***^	0.909^***^	1.627^***^
	(15.394)	(5.970)	(9.350)	(4.967)	(6.640)	(3.161)	(4.032)
R-squared	0.244	0.387	0.467	0.425	0.456	0.476	0.486
r2_a	0.228	0.372	0.454	0.411	0.441	0.462	0.471
*F*	12.834	25.116	30.895	29.364	29.517	31.993	29.910

***p < 0.01, **p < 0.05, *p < 0.1; t-statistics in parentheses.

Second, we examined the mediating role using the bootstrap method (98). The results are presented in Table 7. The variables PC and EE significantly mediate the relationship between AI and CWB, with effect coefficients of 0.036 and 0.083, respectively. Moreover, neither of these coefficients included 0 within the 95% confidence interval. H2 and H3 were thereby supported. The combined mediating influence of PC and EE as a chain-mediating role was also significant, as indicated by the 95% confidence interval that excludes 0. This indicates a sequential mediating influence that accounts for 31.5% of the overall association. H4 was thereby supported.

**Table 7:** Results of the mediation model.

Paths	Effect	SE	LLCI	ULCI
AIA → PC → CWB	0.036	0.016	0.005	0.069
AIA → EE → CWB	0.083	0.021	0.041	0.125
AIA → PC → EE → CWB	0.022	0.008	0.009	0.039
Total effect	0.448	0.033	0.383	0.512
Direct effect	0.307	0.039	0.230	0.384
Indirect effect	0.141	0.027	0.087	0.195

t-statistics in parentheses, ***p < 0.01, **p < 0.05, *p < 0.1.

## 5 Discussion and conclusions

### 5.1 Theoretical contributions

This study is a contribution to the literature in several ways. First, our study extends the application scenarios of the COR theory. AI awareness is linked to CWB through PC and EE. The relationship between AI awareness and CWB is explained from the perspective of resource consumption and conservation. Our study continues the existing research on the associations between AI awareness and employee behaviors, such as turnover intention (99), job insecurity (58), job burnout (100), and job withdrawal (14). Few researchers pay attention to CWB. We extend the research on the impact of AI on employees’ behavioral consequences from the perspective of CWB, thus further validating the negative impact of AI on employees (2, 14, 100). Additionally, numerous previous studies have investigated the antecedents of CWB, including factors such as employee personality, motivation, leader behavior, and internal relationships (35, 39, 42, 43). However, few of these studies have explored the impact of AI awareness on CWB. Thus, this study enriches the CWB literature from different perspectives.

Second, it enriches the literature on the negative impact of AI awareness on employee behavior, particularly during human intelligence collaboration. In contrast to the pervasiveness of AI, research on AI in management is still in its early stages (15). Existing research on AI’s adverse psychological and behavioral effects on employees primarily focuses on the service industry, specifically the hospitality sector. Less attention has been paid to other industries that have more contact with AI. Moreover, most of these affected behaviors are independent and not placed in the context of collaboration with AI. This study examines the overall impact of AI awareness across industries with high AI exposure. Furthermore, it contextualizes the situation within the process of human intelligence collaboration, which echoes Teng et al. (14) call for future research on AI awareness to investigate additional industries for more general conclusions.

Third, combining COR theory with PC theory can help us to understand the changes in employees’ PC and their correlations with behaviors (101). By examining the mediating roles of PC and EE, this paper provides a resource conservation and PC perspective to elucidate the mechanism underlying the relationship between AI awareness and CWB. The current research on the mediating effects of AI awareness on employee behavior is still in its initial stages (58). it expands the exploration of process mechanisms that connect AI awareness to employee work outcomes. Previous studies have confirmed EE’s positive facilitation of CWB (73). However, an associated discussion is needed regarding the increasingly widespread use of AI. Whether the influence of AI awareness on CWB can be mediated by PC and EE has not been conducted. So, we draw on COR theory to investigate the mechanisms of AI awareness on CWB in light of the loss of resources associated with PC and EE, strengthening our comprehension of the underlying mechanisms of how AI awareness influences employee attitudes and behaviors. Previous studies have focused on AI awareness and its impact on employee behaviors, often examining the role of a single mediator or several parallel mediators. However, there has been a lack of in-depth research into the mediation mechanisms of multiple successive mediators in the relationship between AI and CWB. In this paper, we introduce the PC-EE chain mediation model. In this model, both PC and EE can individually act as mediators and they also chain mediate the relationship between AI awareness and CWB. Therefore, our research expands the understanding of the relationship between AI awareness and CWB and contributes to the existing literature by enriching the understanding of AI’s influence on employee behavior through mediation mechanisms.

### 5.2 Implications for practice

In the AI-driven era of Industry 4.0, this study has practical implications for managers who aim to contribute to SDG8. First, as AI becomes increasingly prevalent in manufacturing, service, healthcare, and other industries (102), employees are encouraged to actively embrace, engage with, and utilize AI to maximize its benefits. However, research indicates that AI awareness can negatively impact employees’ emotions. Thus, leaders should focus on facilitating the acceptance of AI among their employees. Managers should address some workers’ high psychological insecurity toward AI adoption. Effective communication regarding the AI implementation process and its potential effects on employees is crucial to reducing misconceptions and alleviating unnecessary AI-associated threats. On the other hand, managers must adopt necessary measures to safeguard the well-being of employees, particularly vulnerable groups such as disabled workers and those with special needs (59, 103).

Second, considering the substantial role of EE in CWB, leaders need to be attentive to their staff’s psychological well-being. Organizations can implement various strategies to mitigate negative emotions such as EE. For instance, providing adequate free time and rest, as mentioned in SDG8, encourages employees to seek a work-life balance and helps reduce feelings of resource depletion and stress. Furthermore, organizations should recognize their employees’ diverse backgrounds and needs (104) and offer necessary psychological counseling services to assist in reducing the impact of negative emotions. Additionally, companies can collaborate with employees to map out their career paths and explore the role of AI technology in career development, enabling employees to recognize the benefits AI brings to their careers. It is also beneficial to assist them in adapting to new technologies to counteract the threat of replacement, thereby upgrading their skill sets.

Third, full attention should be given to the level and fluctuating impact of employees’ PC in the context of AI adoption. Managers can gauge the state of employees’ PC by observing their behavior. Therefore, managers should engage in private, informal conversations with employees to understand the actual status of their PC. At the same time, managers should effectively communicate with employees about the scope, stage, and specific impact of AI adoption. By doing so, leaders can prevent scenarios where loyal employees with strong PC experience a heightened sense of breach due to information asymmetry.

AI adoption affects employees’ perceptions of their jobs and raises concerns about their future careers. Organizations must be vigilant about such situations emerging to address and mitigate employees’ negative emotions and behaviors. Ultimately, employees should be able to work decently and have opportunities for growth and development within the organization.

### 5.3 Strengths

The strengths and contributions of this study are as follows. On the one hand, we enriched the literature on AI awareness by examining the relationship between AI and CWB, identifying CWB as an important behavioral outcome of AI awareness that has not yet been adequately studied. On the other hand, this study explains the mechanisms of how AI awareness affects employees’ CWB by providing a theoretical framework that includes sequential mediating variables. PC and EE sequentially play a mediating role in the association between AI awareness and CWB, thereby enriching research into how AI influences the dark side of employees’ psychology and behavior.

### 5.4 Limitations and future research

Several limitations need to be addressed in future research. First, since our data were collected solely from employees, this could have affected the reliability of the results. Consequently, we recommend that future studies should include a diverse range of sources, such as managers, peers, subordinates, and labor unions, to enhance the reliability of the findings. Second, we identified partial mediators between AI awareness and CWB, indicating that other mediating pathways might influence these two variables. Therefore, we suggest exploring other potential mediators, such as job insecurity and emotional commitment, to enhance AI-related research. Third, this paper does not explore the boundary conditions for the relationship between AI awareness and CWB. In future investigations, potential moderating variables, particularly those that could mitigate the negative consequences of AI awareness, should be investigated. When it comes to selecting validation methods, the results of the hypotheses can be further substantiated using a variety of approaches, including SEM, to bolster the robustness of the findings. The research findings reveal the dark side of AI. Nevertheless, the influence of AI on employees is complex and varied. To date, a considerable body of research has focused on the benefits of AI-customer interactions. Future research can draw upon existing findings from customer-AI interactions to explore the potential positive outcomes and impacts that such interactions could have on organizations and employees. This would contribute to a more comprehensive understanding of the impact of AI on organizations and employees.

### 5.5 Conclusion

This research employs the COR theoretical framework to examine the association between AI awareness and negative employee behavior. Our study suggests that AI awareness is associated with CWB via PC and EE. AI awareness due to AI adoption hampers organizations’ pursuit of SDG8-Decent Work to a certain degree. Consequently, this study is vital for comprehending the negative associations between AI awareness and employee welfare, as well as the relevance of these findings for practical management strategies.

## Data Availability

The raw data supporting the conclusions of this article will be made available by the authors, without undue reservation.
